# Reducing the Carbon Footprint of Neovascular Age–Related Macular Degeneration Therapy: Environmental Impact of Transitioning to Aflibercept 8 mg

**DOI:** 10.1155/joph/9131955

**Published:** 2026-02-23

**Authors:** Daniele Veritti, Micaela Misciagna, Cristian Teatino, Chiara Pontoni, Marianna Blasutig, Mattea Kuftic, Sofia Pece, Andrea Morsanutto, Alfredo Pece, Paolo Lanzetta

**Affiliations:** ^1^ Department of Medicine-Ophthalmology, University of Udine, Udine, Italy, uniud.it; ^2^ Gesteco Spa, Povoletto, Udine, Italy; ^3^ Innovation and Change, Milan, Italy; ^4^ SOC Politiche del Farmaco e Governo dei Percorsi di Appropriatezza, Azienda Sanitaria Universitaria Friuli Centrale, Udine, Italy; ^5^ Fondazione Retina 3000, Milan, Italy; ^6^ Istituto Europeo di Microchirurgia Oculare—IEMO, Udine, Milan, Italy

**Keywords:** aflibercept 8 mg, anti-VEGF therapy, carbon footprint, environmental sustainability, intravitreal injections, neovascular age–related macular degeneration

## Abstract

**Purpose:**

To quantify and compare the carbon footprint associated with neovascular age–related macular degeneration (nAMD) treatment using real‐world intravitreal aflibercept 2‐mg injections versus a simulated aflibercept 8‐mg regimen with extended dosing intervals.

**Methods:**

A life cycle assessment (LCA) combining process‐based and input–output methodologies was conducted using real patient data from a tertiary ophthalmic center. The analysis included 101 patients treated with aflibercept 2 mg and modeled the environmental impact of transitioning to aflibercept 8 mg. The simulated aflibercept 8‐mg cohort was generated by replicating the demographic and logistical characteristics of the real‐world aflibercept 2‐mg population while applying extended dosing intervals. Emissions were estimated in CO_2_ equivalents (CO_2_e) and included drug manufacturing, clinical materials, patient transportation, energy use, and waste disposal. Emission factors were derived from Defra and ecoinvent databases.

**Results:**

Patients treated with aflibercept 2 mg generated an average carbon footprint of 1150 kg CO_2_e per treatment cycle (2 years), compared to 746 kg CO_2_e for those modeled to receive aflibercept 8 mg. This corresponds to an absolute reduction of 404 kg CO_2_e and a relative reduction of 35%. The main driver of emissions was pharmaceutical procurement, followed by patient transportation. The lower injection frequency enabled by aflibercept 8 mg significantly reduced both sources of emission.

**Conclusions:**

A transition from conventional aflibercept 2‐mg therapy to an aflibercept 8‐mg regimen with extended dosing intervals reduces the treatment’s carbon footprint by over one‐third. These findings support the integration of environmental considerations into therapeutic decisions in ophthalmology, particularly for high‐frequency procedures, such as intravitreal injections.

## 1. Introduction

The healthcare sector is responsible for approximately 4%–10% of global greenhouse gas emissions in developed countries, potentially offsetting the very health benefits it is intended to provide [1, 2]. As climate change increasingly threatens global health, reducing the environmental footprint of healthcare delivery has become an urgent priority while preserving or improving clinical outcomes [3]. However, sustainability in health care lacks a universally accepted definition, and carbon footprint analysis has emerged as a pragmatic and widely adopted metric to quantify environmental impact across healthcare systems [4, 5]. Ophthalmology, particularly in the management of chronic conditions requiring frequent interventions, presents both challenges and opportunities for environmental sustainability. Neovascular age–related macular degeneration (nAMD) is a leading cause of vision loss among older adults in developed countries, affecting over 196 million people globally [6]. The standard of care for nAMD involves intravitreal anti–vascular endothelial growth factor (anti‐VEGF) injections, with aflibercept being one of the most widely used agents, accounting for a substantial proportion of anti‐VEGF treatments in routine clinical practice worldwide [7–10]. These treatments have dramatically improved visual outcomes but typically require frequent administrations over multiple years, creating a substantial treatment burden for patients, healthcare systems, and the environment, particularly due to repeated clinical visits, pharmaceutical supply chains, and associated resource consumption [10–15].

Intravitreal injections (IVIs) have become the most common ophthalmic procedure worldwide, with an estimated 15 million injections performed annually in the United States alone [16]. This volume is expected to increase further as population aging drives a rising prevalence of chronic retinal diseases, such as nAMD. The environmental impact of IVIs has therefore attracted increasing attention, with recent position papers and sustainability initiatives highlighting the substantial contribution of packaging, transport, storage, and waste generated by high‐volume retinal care [16–19]. Recent studies quantify the carbon footprint of a single IVI at approximately 13.7–14.1 kg CO_2_ equivalent (CO_2_e), excluding the anti‐VEGF medication itself [2, 20]. Importantly, published estimates vary widely depending on the life cycle assessment (LCA) boundaries adopted, ranging from procedure‐only analyses to factory‐gate‐to‐patient or full care‐pathway approaches [5, 21]. The main sources of these emissions include patient travel (accounting for approximately 77% of emissions), procurement of medical supplies (19%), and building energy use (4%) [2, 20]. Consistent findings across different healthcare systems confirm patient travel and pharmaceutical supply chains as dominant emission drivers in intravitreal therapy pathways [2, 5, 20].

Demographic trends further amplify this challenge. As global life expectancy continues to rise, the prevalence of age‐related conditions, such as nAMD, is projected to increase substantially, leading to a higher cumulative demand for IVIs, healthcare resources, and associated medical waste. This anticipated burden has driven growing interest in therapeutic strategies aimed at reducing injection frequency while maintaining clinical efficacy, thereby addressing both patient burden and environmental sustainability. The introduction of higher‐dose aflibercept (8 mg) offers the potential to reduce treatment burden through less frequent dosing while maintaining comparable efficacy to the standard 2‐mg formulation [22, 23]. Aflibercept 8 mg is a concentrated formulation designed to deliver a fourfold higher molar dose compared with aflibercept 2 mg, enhancing intravitreal durability through high VEGF‐binding affinity and a prolonged intraocular half‐life [24]. Recent clinical trials have demonstrated that aflibercept 8 mg can extend treatment intervals while providing noninferior visual outcomes compared to aflibercept 2 mg [25]. This evolution in therapeutic approach creates an opportunity to examine not only clinical and quality‐of‐life benefits but also potential environmental advantages.

While the primary goal of medical innovation remains improving patient outcomes, the environmental implications of treatment regimens are increasingly relevant considerations in healthcare decision‐making. To date, only a limited number of studies have quantitatively assessed the environmental impact of ophthalmic treatments, particularly regarding the carbon footprint of varying anti‐VEGF regimens in nAMD management. Moreover, most available analyses focus on per‐injection estimates rather than patient‐level, longitudinal treatment pathways. As such, there remains a clear gap in understanding the cumulative environmental impact of treatment strategies over clinically meaningful time horizons.

In this study, we analyze real‐world data from 101 nAMD patients treated with aflibercept 2 mg over a two‐year period to estimate and compare the carbon footprint of conventional aflibercept 2 mg versus the higher‐dose aflibercept 8‐mg regimen. By measuring actual injection frequencies, clinical visits, transportation modes, and resource utilization, and adopting a comprehensive care‐pathway perspective, we aim to provide a comprehensive assessment of the potential environmental benefits that might be achieved through treatment optimization with higher‐dose aflibercept.

## 2. Methods

### 2.1. Study Design

This retrospective observational comparative study evaluated the carbon footprint of two intravitreal anti‐VEGF treatment regimens for nAMD: a real‐world cohort receiving aflibercept 2 mg according to current clinical practice (fixed bimonthly regimen following 3 monthly loading doses as per VIEW trials) [26] and a simulated cohort receiving high‐dose aflibercept 8 mg administered according to extended dosing intervals modeled on the PULSAR trial [25]. The environmental impact was assessed in terms of carbon dioxide equivalent (CO_2_e) emissions over a two‐year treatment horizon. Emission sources included patient transport, clinical resource use, medical waste generation, drug procurement, and facility energy consumption. The study was conducted at a tertiary referral center in Italy (Department of Ophthalmology, University of Udine, Udine, Italy). The study adhered to the tenets of the Declaration of Helsinki, and institutional review board approval was obtained.

### 2.2. Patients

Two cohorts of 101 patients each were analyzed. The real‐world cohort included consecutive treatment‐naïve patients with newly diagnosed nAMD who initiated aflibercept 2‐mg therapy at our tertiary referral center in 2022. Inclusion criteria were as follows: (1) treatment‐naïve patients with a new diagnosis of nAMD; (2) initiation of intravitreal aflibercept 2‐mg therapy at our institution; (3) availability of complete clinical and follow‐up data for a minimum of 24 months; and (4) availability of residential address information to allow estimation of travel‐related emissions. Exclusion criteria included the following: (1) previous treatment with any intravitreal anti‐VEGF or corticosteroid therapy; (2) concomitant retinal diseases requiring IVIs (e.g., diabetic macular edema or retinal vein occlusion); (3) participation in interventional clinical trials during the study period; and (4) incomplete clinical, logistical, or follow‐up data. Each patient was followed for a 24‐month period. Data were extracted from electronic medical records and supplemented through standardized patient interviews. For each patient, we recorded the total number of clinic visits, residential address, mode of transportation, and whether the patient was accompanied. Transportation modes were categorized as private car, public transport, taxi, walking/cycling, or other. The simulated aflibercept 8‐mg cohort was generated by replicating the demographic and logistical profiles of the real‐world patients, including residential location, transportation mode, and accompaniment. The only variable modified was the number of injections, which was adjusted according to the treatment intervals reported in the PULSAR trial. PULSAR q16 injection frequency heatmap analyses were used to derive interval‐maintenance proportions over time. These proportions were applied to the simulated cohort following an initial three‐dose monthly loading phase [25].

### 2.3. Intravitreal Injection Procedures

Intravitreal aflibercept injections were performed according to a standardized protocol routinely used at our institution. For the real‐world aflibercept 2‐mg cohort, patients received three initial monthly loading injections followed by fixed bimonthly dosing, in accordance with the VIEW trial regimen. Each injection visit included patient check‐in, visual acuity assessment, IVI performed under sterile conditions, and postprocedure monitoring. In the simulated aflibercept 8‐mg cohort, the IVI procedure itself was assumed to be identical to that of the 2‐mg regimen in terms of clinical setting, materials used, staff involvement, and waste generation per injection. The only modeled difference between the two groups was the number of injections and clinic visits over the two‐year period, reflecting the extended dosing intervals associated with aflibercept 8 mg. No additional procedures or visits were assumed for the 8‐mg cohort beyond those directly related to injection administration.

### 2.4. Carbon Footprint Assessment

The carbon footprint for each patient was calculated as the total sum of greenhouse gas emissions associated with patient transportation, pharmaceutical procurement, clinical materials and waste, and facility energy use over a two‐year treatment period. Emissions were expressed as kilograms of carbon dioxide equivalents (kg CO_2_e) per patient. Formally, total emissions were calculated as follows:

(1) Total CO2e=CO2etransport+CO2epharmaceuticals+CO2ematerials+waste+CO2eenergy,

where each component represents the cumulative emissions generated across all clinic visits and IVIs during the treatment cycle.

#### 2.4.1. Patient Transportation (CO_2_e_Transport)

Transport‐related emissions were calculated at the individual patient level by multiplying the round‐trip distance between each patient’s residence and the treatment facility by the total number of clinic visits and by mode‐specific emission factors. Residential locations were geocoded to estimate travel distances, and transportation modes (e.g., private car, public transport, taxi, and walking/cycling) were recorded for each patient, including whether the patient was accompanied. Emission factors per kilometer for each transportation mode were obtained from the ecoinvent database and applied consistently across both treatment scenarios [27].

#### 2.4.2. Pharmaceutical Procurement (CO_2_e_Pharmaceuticals)

Emissions associated with intravitreal drugs and concomitant topical medications were estimated using an economic input–output LCA approach. Specifically, total pharmaceutical expenditure per patient was linked to standardized Defra emission factors, expressed as kilograms of CO_2_e per euro of expenditure [28]. This method captures upstream emissions related to drug manufacturing, packaging, and distribution without modeling each production step individually and provides a pragmatic and reproducible estimate of pharmaceutical‐related emissions in the absence of product‐specific life cycle inventories.

#### 2.4.3. Clinical Materials and Waste (CO_2_e_Materials + Waste)

Emissions from clinical materials were estimated using a process‐based LCA. All disposable materials used during a standard intravitreal aflibercept injection at our institution were individually identified and weighed, including syringes, needles, drapes, gloves, gauze, packaging components, and ancillary consumables. Materials were categorized by material type (e.g., plastics, paper, and metals) and multiplied by mass‐based emission factors derived from the ecoinvent database. No reusable devices were employed in the IVI procedure. End‐of‐life treatment of clinical waste was modeled as incineration, in accordance with routine hospital waste management practices. The same set of materials per injection was assumed for both aflibercept 2‐ and 8‐mg regimens, with differences in total emissions arising solely from the number of injections performed [27].

#### 2.4.4. Facility Energy Use (CO_2_e_Energy)

Energy‐related emissions associated with clinic operations were estimated based on national benchmarks for hospital electricity consumption. Energy use was adjusted for the proportion of clinical space and time allocated to IVI activities. Electricity‐related emissions were converted to CO_2_e using the Italian electricity emission factor provided by the Association of Issuing Bodies (AIB) [29].

Overall, the analysis included upstream (manufacturing and procurement), use‐phase (clinical activities and patient transportation), and end‐of‐life (waste disposal) life cycle stages. Capital equipment, infrastructure construction, and staff‐related emissions were excluded from the system boundaries.

A sensitivity analysis was conducted to assess the robustness of the results under varying assumptions, including changes in average travel distance, transportation mode distribution, and energy intensity, while maintaining all other parameters constant.

### 2.5. Statistical Analysis

The primary outcomes of the analysis were the total CO_2_e emissions per patient over the 2‐year treatment cycle, the relative contributions of each emission source, and the potential emission reductions achievable with extended‐interval therapy. Summary statistics were reported as means ± standard deviation (SD). Emission reductions were expressed in both absolute (kg CO_2_e) and relative (%) terms. Normality of distribution was assessed using the Shapiro–Wilk test. Depending on the distribution of data, comparisons between the two treatment regimens were performed using either the paired Student’s *t*‐test (for normally distributed data) or the Wilcoxon signed‐rank test (for non‐normally distributed data). A two‐tailed *p* value <  0.05 was considered statistically significant. All analyses were performed using IBM SPSS Statistics, Version 29.0 (IBM Corp., Armonk, NY, USA).

## 3. Results

### 3.1. Patient Characteristics

A total of 101 patients receiving standard therapy with aflibercept 2 mg (real‐world cohort, AS‐IS scenario) and 101 matched patients modeled to receive aflibercept 8 mg (TO‐BE scenario) were included in the analysis. The mean age was 76.8 ± 7.3 years, with a slight female predominance (58%). The average round‐trip distance from patients’ residences to the clinic was 102.3 ± 90.1 km (range: 5.2–494.0 km). Private vehicles were the most common mode of transportation (64%), followed by public transportation (29%), taxi services (5%), and other modes. Most patients (72%) were accompanied by a caregiver or family member to their appointments.

### 3.2. Treatment Frequency

In the real‐world cohort, patients received a mean of 12.66 ± 0.99 injections over two years. In the modeled aflibercept 8‐mg cohort, patients received an average of 8.22 ± 1.35 injections over two years (*p* < 0.001).

### 3.3. Carbon Footprint Analysis

**FIGURE 1:**
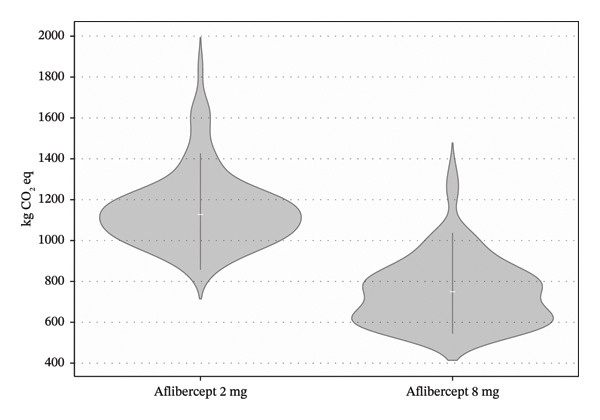
Distribution of carbon footprint per patient by group. Violin plot illustrating the distribution of total carbon emissions (kg CO_2_ e) per patient over a 2‐year treatment cycle, comparing aflibercept 2 mg (blue) and aflibercept 8 mg (orange). The width of each shape represents the kernel density of the data, while the white line indicates the median, with thick bars corresponding to the interquartile range. Aflibercept 8 mg is associated with a significantly lower carbon footprint.

The average total carbon footprint per patient over the two‐year treatment cycle was 1150 ± 193 kg CO_2_e in the aflibercept 2‐mg cohort and 746 ± 144 kg CO_2_e in the aflibercept 8‐mg cohort, resulting in an absolute reduction of 404 kg CO_2_e and a relative reduction of 35.1%. This difference was statistically significant (paired *t*‐test, *p* < 0.001) (Figure 1).

**FIGURE 2:**
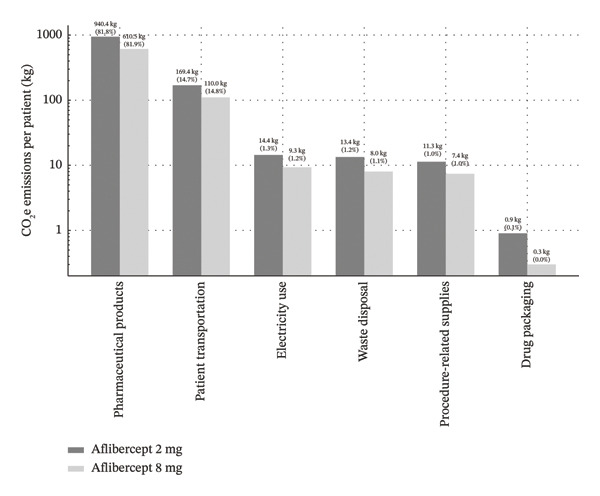
Average CO_2_e emissions per patient over a 2‐year treatment cycle: Aflibercept 2 vs 8 mg bar chart comparing the average carbon footprint (in kg of CO_2_ e) per patient over a 2‐year treatment cycle for intravitreal aflibercept 2 versus 8 mg regimens. Emissions are broken down by source: pharmaceutical product manufacturing, patient transportation, electricity use, waste disposal, procedure‐related supplies, and drug packaging. High‐dose, 8 mg aflibercept results in substantially lower total emissions (746 kg CO_2_e vs. 1,150 kg CO_2_e). Data are presented on a logarithmic scale to enhance the visibility of minor emission sources. Values represent absolute emissions and their percentage contribution to the total.

The pharmaceutical component accounted for the largest share of emissions, contributing approximately 82% of the total carbon footprint. Patient transportation was the second‐largest contributor. The reduction in injection frequency with aflibercept 8 mg resulted in fewer clinic visits and, therefore, a proportional decrease in transportation‐related emissions. A detailed breakdown of emissions by source is presented in Table 1 and Figure 2. When expressed on a per‐injection basis, the mean overall carbon footprint (including pharmaceutical‐related emissions) was 90.83 kg CO_2_e per IVI in the aflibercept 2‐mg cohort and 90.75 kg CO_2_e per injection in the modeled aflibercept 8‐mg cohort. When excluding the pharmaceutical component, the procedural carbon footprint was 16.35 kg CO_2_e per IVI.

**TABLE 1:** Average CO_2_e emissions per patient over a 2‐year treatment cycle, by emission source.

Category	Aflibercept 2 mg	Aflibercept 8 mg
Average number of injections	12.66	8.22
Pharmaceutical products	940.4 kg CO_2_e	610.5 kg CO_2_e
Patient transportation	169.4 kg CO_2_e	110.0 kg CO_2_e
Electricity use	14.4 kg CO_2_e	9.3 kg CO_2_e
Waste disposal	13.4 kg CO_2_e	8.0 kg CO_2_e
Procedure‐related supplies	11.3 kg CO_2_e	7.4 kg CO_2_e
Drug packaging	0.9 kg CO_2_e	0.3 kg CO_2_e
Total	1150 kg CO_2_e	746 kg CO_2_e

### 3.4. Sensitivity Analysis

A sensitivity analysis was performed to evaluate the robustness of results under varying assumptions regarding transportation and clinical waste emissions. When varying the average transportation‐related emissions by ± 50%, the total difference in carbon footprint between the aflibercept 2‐mg and 8‐mg regimens ranged from 367.2 kg CO_2_e (−9.0%) to 439.9 kg CO_2_e (+9.0%), compared to the baseline difference of 404 kg CO_2_e. This confirms that patient travel, while impactful, does not alter the primary outcome of substantial emission reduction with the extended‐interval aflibercept 8‐mg regimen. Similarly, when emissions associated with clinical waste were varied by ±50%—reflecting variations in the quantity and composition of disposable materials and associated packaging used per IVI (e.g., syringes, needles, sterile drapes, gloves, gauze, and packaging components)—the difference in total emissions ranged from 393.5 kg CO_2_e (−2.5%) to 413.6 kg CO_2_e (+2.5%). This indicates a relatively minor effect, consistent with previous findings that medical waste represents a small fraction of the total carbon footprint of intravitreal procedures. Overall, the environmental advantage of the aflibercept 8‐mg regimen remained significant and stable across all modeled scenarios.

### 3.5. Projected Population‐Level Impact

Extrapolating the observed emission reductions to a population level highlights the potential environmental benefit of extended‐interval therapy. In the European Union, nAMD is estimated to affect approximately 1.7 million individuals, and the annual incidence of late AMD exceeds 170,000 new cases per year; given that nAMD is reported to be about 1.7 times more common than geographic atrophy, this corresponds to roughly ∼100,000 newly incident nAMD cases annually (i.e., ∼200,000 over a two‐year horizon) [30–32]. Using this conservative two‐year incident cohort as an illustrative scenario (estimate to be interpreted as an order‐of‐magnitude approximation rather than a precise epidemiological count), a per‐patient reduction of 404 kg CO_2_e over two years would translate into an aggregate reduction of approximately 80,000 metric tons of CO_2_e per two‐year treatment cycle by transitioning from first‐generation, high‐frequency anti‐VEGF regimens to aflibercept 8 mg, within routine clinical practice.

## 4. Discussion

This study demonstrates that transitioning from standard‐dose aflibercept 2 mg to high‐dose aflibercept 8 mg in the management of nAMD leads to a substantial and statistically significant reduction in carbon dioxide equivalent (CO_2_e) emissions. Over a two‐year treatment cycle, the extended‐interval regimen with aflibercept 8 mg reduced the mean number of IVIs by approximately 35%, resulting in an average per‐patient emission reduction of 404 kg CO_2_e. These findings highlight the substantial environmental benefits associated with adopting next‐generation intravitreal agents—such as high‐dose aflibercept and other durable anti‐VEGF therapies—which enable reduced injection frequency and thereby support the integration of sustainability principles into therapeutic decision‐making in ophthalmology.

The present results are consistent with previous studies estimating the carbon footprint of IVIs. Power et al. calculated a per‐injection carbon footprint of approximately 13.7 kg CO_2_e, excluding the pharmaceutical component, with the majority of emissions attributed to patient travel, followed by procurement of medical supplies and facility energy use [2]. Similarly, Chandra et al. reported a comparable value of 14.1 kg CO_2_e per injection, further validating the robustness of current LCA methodologies applied to intravitreal therapy [20]. In our real‐world analysis, the procedural footprint per injection (excluding pharmaceuticals) aligns closely with these estimates (16.35 kg CO_2_e), supporting their generalizability across different healthcare systems and reinforcing the central role of patient logistics in determining environmental impact [33].

When the pharmaceutical component is included, our findings indicate that drug procurement accounts for approximately 82% of total emissions, reflecting the carbon intensity of pharmaceutical manufacturing, packaging, and upstream supply chains [19]. This observation is consistent with broader healthcare LCA literature, which has repeatedly identified pharmaceuticals as a dominant contributor to healthcare‐related emissions due to energy‐intensive production processes and complex global distribution networks [5, 17, 19]. Our detailed breakdown of emission sources confirms that pharmaceutical production and patient transportation together represent the primary drivers of the total carbon footprint associated with anti‐VEGF therapy, whereas clinical energy consumption and medical waste disposal contributed only marginally to overall emissions. This pattern has been consistently reported across multiple healthcare systems and ophthalmic settings [2, 20, 21].

A recent study by Bowley et al. specifically evaluated the environmental impact of using aflibercept 8 mg instead of aflibercept 2 mg in treatment‐naïve patients with nAMD in the United Kingdom, using a factory‐gate‐to‐patient, per‐injection analytical framework [5]. Although their reported per‐injection emissions (approximately 2.3 kg CO_2_ per injection for aflibercept 2 mg and 2.1 kg CO_2_ for aflibercept 8 mg) are numerically lower than those observed in our study, this difference is primarily explained by methodological scope, as their analysis excluded pharmaceutical manufacturing emissions and focused on packaging, transport, patient travel, and waste disposal. Despite these differences, both analyses converge on the same central conclusion: The principal mechanism through which aflibercept 8 mg reduces environmental impact is the reduction in injection frequency and associated hospital visits, rather than changes in the injection procedure itself. This consistency across distinct methodological approaches strengthens the overall evidence base supporting treatment durability as a key lever for improving sustainability in retinal care.

Extrapolating our findings to the European population with nAMD suggests that widespread adoption of aflibercept 8 mg during the first two years of treatment could result in a reduction of over 80,000 metric tons of CO_2_e, assuming that approximately 200,000 patients are within the early treatment phase [30–32]. Based on standard European emission conversion factors, this reduction is equivalent to removing approximately 9000–10,000 gasoline‐powered passenger vehicles from the road for two years, or to the average electricity consumption of more than 20,000 European households over the same period [5, 34–39]. These comparisons provide an intuitive representation of the magnitude of the environmental benefit while remaining grounded in established emissions accounting methodologies.

Beyond modifications in dosing regimen, additional strategies may further enhance the sustainability of intravitreal therapy pathways. Excessive packaging and reliance on single‐use materials contribute significantly to procedural waste, and several studies have advocated for more compact, recyclable, or circular packaging solutions. Pilot programs exploring the reuse of shipping materials—such as polystyrene coolers and cold packs—have demonstrated substantial reductions in both carbon emissions and landfill volume [16, 40]. Furthermore, evidence suggests that streamlined, low‐consumption IVI techniques can be safely implemented without increasing infection risk, offering additional opportunities to reduce material use in routine clinical practice [41–44].

Despite the strengths of our methodology and the real‐world nature of the aflibercept 2‐mg cohort, several limitations should be acknowledged. The study was conducted at a single tertiary referral center, and the findings reflect local geographic and organizational characteristics. Moreover, while the aflibercept 8‐mg cohort was simulated using treatment interval data derived from published PULSAR trial heatmaps, it does not represent real‐world longitudinal experience with this formulation. Although this modeling approach is consistent with prior sustainability analyses and was designed to reflect clinically realistic dosing patterns, prospective real‐world studies will be essential to confirm these findings as aflibercept 8 mg becomes more widely adopted.

In conclusion, the use of aflibercept 8 mg administered at extended intervals offers a clinically effective and environmentally advantageous alternative to conventional aflibercept 2‐mg therapy. By reducing injection frequency, extended‐interval anti‐VEGF regimens substantially lower the carbon footprint of nAMD care at both the patient and population levels. These findings support the integration of environmental sustainability into therapeutic decision‐making in ophthalmology and highlight the broader potential of transitioning from first‐generation to next‐generation durable anti‐VEGF therapies. Future research should validate these benefits across diverse healthcare systems, refine pharmaceutical life cycle data, and explore system‐level interventions to further reduce the environmental impact of chronic retinal disease management.

## Funding

No funding was received for this manuscript. Open access publishing facilitated by Universita degli Studi di Udine, as part of the Wiley‐CRUI‐CARE agreement.

## Conflicts of Interest

Daniele Veritti is a consultant for AbbVie, Bayer, and Roche.

Paolo Lanzetta is a consultant for AbbVie; Paolo Lanzetta is a consultant for AbbVie, Apellis, Bausch & Lomb, Bayer, Biogen, Boehringer Ingelheim, I‐Care, Genentech, Ocular Therapeutix, Outlook Therapeutics, and Roche.

The other authors declare no conflicts of interest.

## Data Availability

Data are available upon request from the authors.
